# A protracted diagnostic journey of pediatric PAPA syndrome and subsequent response to tofacitinib therapy: a case report and literature review

**DOI:** 10.3389/fimmu.2026.1870454

**Published:** 2026-08-18

**Authors:** Shuien He, Hao Deng, Jinghua Yang, Wenwen Jiang

**Affiliations:** 1 Department of Pediatrics, The Second Affiliated Hospital of Guangzhou University of Chinese Medicine, Guangdong Provincial Hospital of Chinese Medicine, Guangzhou, China; 2 Xiaorong Luo’s National Famous Expert Inheritance Studio, Guangdong Provincial Hospital of Chinese Medicine, Guangzhou, China; 3 Shanghai Jiao Tong University School of Medicine, Shanghai, China; 4 Ying Lv’s Expert Inheritance Studio, Guangdong Provincial Hospital of Chinese Medicine, Guangzhou, China; 5 Youjia Xu’s Famous Expert Inheritance Studio, Guangdong Provincial Hospital of Chinese Medicine, Guangzhou, China; 6 Mingzhao Du’s Inheritance Studio of Chinese Medicine, Guangdong Provincial Hospital of Chinese Medicine, Guangzhou, China

**Keywords:** Asian, case report, JAK inhibitor, p.E257G, PAPA syndrome, pediatric, PSTPIP1, Tofacitinib

## Abstract

PAPA syndrome (pyogenic sterile arthritis, pyoderma gangrenosum, and acne) is a rare autosomal dominant autoinflammatory disorder caused by mutations in the PSTPIP1 gene. In pediatric patients, arthritis often precedes cutaneous manifestations by several years, leading to frequent misdiagnosis as septic arthritis. Treatment is challenging, with IL-1 inhibitors being the most pathophysiology-based first-line therapy, but their accessibility is limited by high costs. We reported an 11-year-old Chinese boy with a 7-year history of recurrent, migratory arthritis. Trio whole-exome sequencing identified a heterozygous PSTPIP1 c.770A>G (p.Glu257Gly) variant, classified as likely pathogenic. The father carried the same variant; he had experienced arthralgias in early childhood but became asymptomatic after age 10 without any treatment, demonstrating incomplete penetrance. This variant is extremely rare, reported in only one of 16 patients in the Eurofever registry, all of European descent, making this the first Asian case. Due to financial constraints, IL-1 inhibitors were inaccessible. Tofacitinib (3.5 mg twice daily, adjusted from adult dose based on body surface area) was initiated in February 2026. After 2 months, joint swelling and pain decreased significantly. As of July 10, 2026, no recurrence of joint swelling and pain had been observed during follow-up. However, radiography revealed irreversible structural damage (bone demineralization, joint space narrowing, osteophyte formation), and the patient still had limited mobility. Rehabilitation specialists advised against aggressive range-of-motion exercises and recommended home-based ultrasound and electrical stimulation to prevent muscle atrophy. Long-term follow-up is ongoing. PAPA syndrome should be considered in children with culture-negative, antibiotic-unresponsive recurrent arthritis, even without skin findings. Early genetic diagnosis can prevent unnecessary procedures. For patients who cannot access IL-1 inhibitors, tofacitinib may reduce inflammation, but it cannot reverse established joint damage. Given the relatively short follow-up duration, the long-term durability of response, safety, and impact of tofacitinib on disease progression remain to be determined. This report provides early, preliminary evidence of JAK inhibitor use in this population, contributing initial data to the limited Asian literature.

## 1 Introduction

PAPA syndrome (OMIM #604416) is a rare autosomal dominant autoinflammatory disorder caused by mutations in the PSTPIP1 gene (1). Its classic clinical triad includes pyogenic sterile arthritis, pyoderma gangrenosum (PG), and acne. The pathophysiology involves hyperactive PSTPIP1 protein, leading to inflammasome activation and excessive IL-1β production (2, 3). However, clinical presentation demonstrates significant heterogeneity, even within families. In pediatric cases, arthritis typically precedes cutaneous manifestations by several years, frequently resulting in misdiagnosis as septic arthritis (4, 5). Recent advances in genetic diagnosis have facilitated earlier recognition of PAPA syndrome. Over 30 sequence variants in PSTPIP1 have been detected in affected individuals, with most pathogenic mutations concentrated in the F-BAR domain, which is critical for membrane localization and protein-protein interactions (6, 7). Genotype-phenotype correlations remain incompletely understood. Demidowich et al. reported that PSTPIP1 mutations are incompletely penetrant and variably expressed, and identified A230T and E250K as disease-associated mutations (8). Certain variants, such as E250K and A230T, have been associated with more severe disease phenotypes. The p.E257G variant identified in our patient is extremely rare. Data from the Eurofever registry, which included 16 PAPA syndrome patients, identified only one patient carrying the E257G mutation, highlighting the rarity of this specific mutation (9).

## 2 Case presentation

### 2.1 Patient history

**Figure 1:**
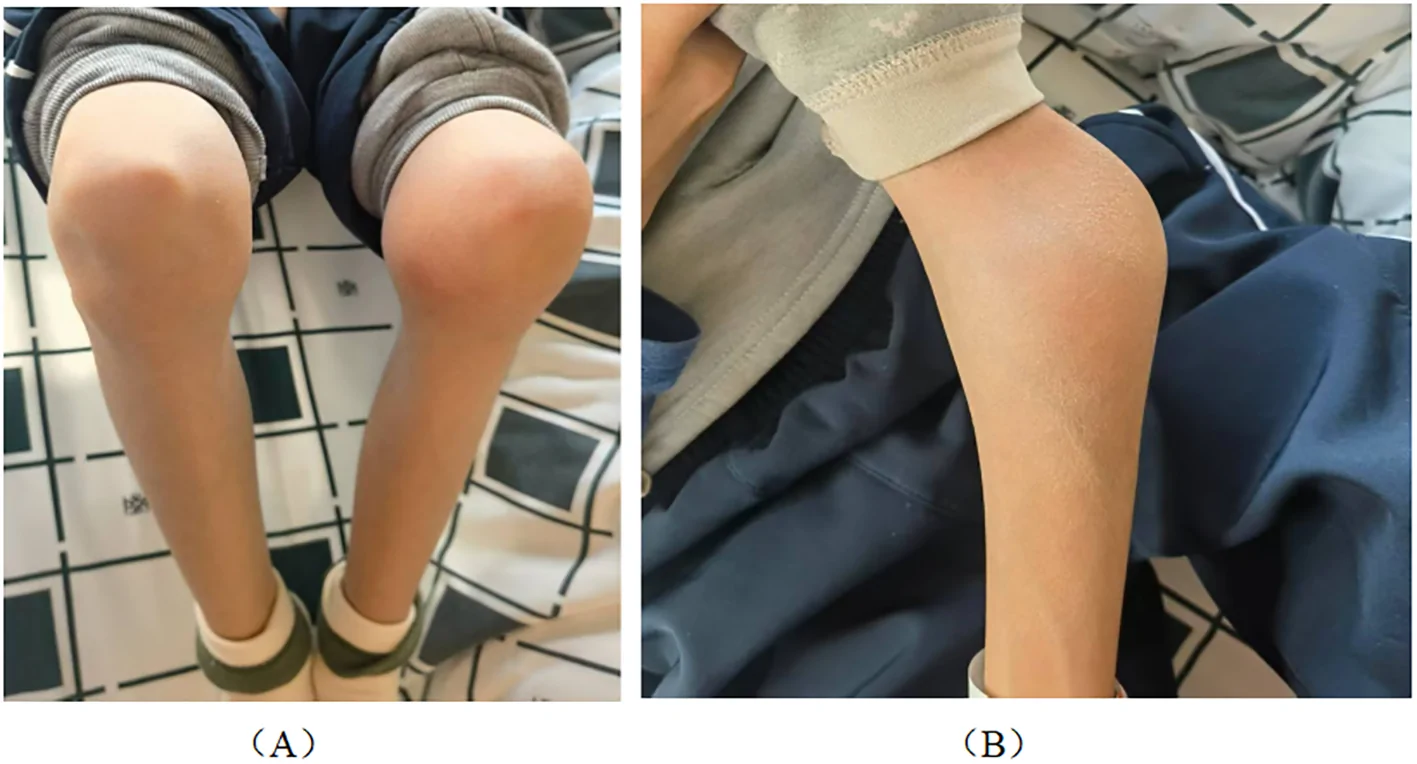
**(A, B)** Clinical photographs of the patient before tofacitinib treatment (February 2026). **(A)** Right knee swelling; **(B)** Left ankle swelling.

An 11-year-old Chinese boy presented with a 7-year history of recurrent, migratory arthritis. **Table 1** summarizes the chronological disease course, key diagnostic procedures, successive therapeutic regimens and serial laboratory and imaging examinations. In 2018, at age 4, he developed right elbow swelling and pain after minor trauma. Radiographs were unremarkable. He underwent three surgical debridements between 2018 and 2021 for suspected septic arthritis; all intraoperative cultures were negative. Multiple courses of antibiotics failed to control his symptoms. The arthritis subsequently involved the right knee and ankle. Rheumatoid factor, anti-CCP, and ANA were negative. Tests for tuberculosis and other microbial workups were negative. Cytokine analysis showed that the levels of TNF-α, IL-1β, IL-4, IL-5, IFN-α, and IL-12p70 were all within the reference ranges. IL-6 level was mildly increased to 39.80 pg/mL (reference range, 0–7 pg/mL). A contrast-enhanced MRI of the right knee (December 5, 2020) demonstrated joint effusion, marked synovial thickening and enhancement, suggestive of pyogenic arthritis, with perisynovial soft-tissue edema. Pre-tofacitinib MRI of the left elbow and knee (February 7, 2026) revealed marked synovial thickening encasing the bones and ligaments, juxta-articular bone signal abnormalities (hyperintensity on fat-suppressed T2WI) with relatively preserved morphology, prominent soft-tissue swelling, and small joint effusions. Scattered round T1-hypointense, fat-suppressed T2-hyperintense foci were noted around the left elbow; the left popliteal fossa showed multiple well-defined round PD-FS-hyperintense, T1-hypointense lesions (largest 1.0 cm). In conjunction with the clinical history, these findings are consistent with PAPA syndrome. **Figure 1** showed clinical photographs of the patient before tofacitinib treatment.

**Table 1:** Chronological summary of disease course, key diagnostic events, sequential treatments, and laboratory and imaging examinations.

Time (Date)	Clinical event	Laboratory and imaging examinations	Treatment (Dose & Duration)	Response
Jul 2018	Right elbow swelling, pain, and low-grade fever after a fall	subsequent DR at a specialized orthopedics hospital: right humeral supracondylar fracture	cast immobilization for 2 weeks	No improvement in swelling, pain, or fever
Sep 2018	Referred to a tertiary hospital	WBC 9.2–11.6×10^9^/L, predominantly neutrophils; CRP 32–102 mg/L; ESR 59–92 mm/h	Arthroscopic debridement of right elbow; postoperative cefoperazone-sulbactam plus clindamycin	Postoperative ESR and CRP improved temporarily, but joint swelling and pain did not resolve
Oct 2018	Transferred to a tertiary hospital	ESR 87 mm/h, WBC 11.09×10^9^/L, CRP 82.3 mg/L; Elbow ultrasound: ill-defined mixed-echo mass (44×35×31 mm) with multiple irregular fluid collections	Open debridement and drainage for pyogenic arthritis of right elbow twice, followed by focal debridement and closure; oral linezolid added	Intermittent fever persisted during antibiotic course; symptoms partly subsided
Jul 2019	Spontaneous right ankle swelling, pain, and low-grade fever	Right ankle MRI: abnormal signal in tibialis anterior, flexor hallucis longus, and extensor digitorum brevis muscles; joint effusion; perisynovial soft-tissue edema, suggestive of inflammation	Ibuprofen and physical therapy	Fever and arthralgia partly resolved
Jun 2020	Bilateral lower limb pain, low-grade fever, inability to stand or walk	WBC 12.02×10^9^/L, Hb 112 g/L, PLT 442×10^9^/L, CRP 6.34 mg/L, ESR 61 mm/h	Prednisone (dose unknown) for anti-inflammation, inosine for 1 week	Symptoms improved, and the patient was discharged
Dec 2020	Right knee swelling, pain, and fever after a fall	Contrast-enhanced MRI of right knee showed joint effusion, marked synovial thickening and enhancement, suggestive of septic arthritis, with surrounding soft-tissue edema	Vancomycin plus azithromycin IV for 4 days	No significant improvement
Jan 2021 - Jul 2022	Recurrent migratory limb joint swelling, pain, and low-grade fever every 3 - 4 months	Normal blood tests, biochemistry, coagulation, HLA-B27, bone marrow aspiration, EBV/CMV-DNA unremarkable; whole-exome sequencing revealed a heterozygous PSTPIP1 mutation	No specific treatment initiated	Spontaneous resolution between episodes
Apr 2023	Admitted to other tertiary hospital; formal diagnosis of PAPA syndrome	Diagnosis confirmed based on clinical and genetic data	Celecoxib for pain, oral methotrexate (dose unknown) + folic acid, and adalimumab (every 2 weeks)	Initial reduction in the frequency of migratory arthralgia, but after two doses of adalimumab, attacks became more frequent; parents discontinued therapy and switched to traditional Chinese medicine
Jun 2024 - Aug 2025	Local hospital: regimen adjustment	NA	Weekly oral methotrexate 5 mg + folic acid 10 mg, meloxicam 5.625 mg qd, prednisolone acetate 2.5 mg qd, combined with traditional Chinese medicine; Methotrexate, folic acid, and meloxicam: discontinued on 07 February 2025; prednisolone reduced to 0.83 mg qod by 31 August 2025	Initial improvement of arthralgia followed by recurrence, with swelling, warmth and tenderness of left elbow and knee
Feb 2026	Admitted for systematic treatment; initiation of tofacitinib	Pre-treatment MRI: left elbow and knee synovial thickening, bone signal abnormalities, effusion, popliteal cysts; Baseline: hs-CRP 45.2 mg/L (reference range, 0 - 6 mg/L), ESR >120 mm/h (reference range,0 - 15 mm/h), TNF-α 3.4 pg/ml (reference range, ≤ 8.2 pg/ml), IL-1β 2 pg/ml (reference range, ≤ 5 pg/ml), IL-4 <3 pg/ml (reference range, ≤ 10 pg/ml), IL-5 <2.5 pg/ml (reference range, ≤ 5 pg/ml), IFN-α <2 pg/ml (reference range, ≤ 10 pg/ml), IL-12p70 <2 pg/ml (reference range, ≤ 4 pg/ml), WBC 7.46×10^9^/L (reference range, 4.3 - 11.30), NEUT% 49.8% (reference range, 31% - 70%), LYM% 42.9% (reference range, 23% - 59%), Hb 116 g/L (reference range, 118 - 156 g/L), PLT 560×10^9^/L (reference range, 167 - 453); 2-month follow-up: WBC 5.51×10^9^/L, NEUT% 31.3%, LYM% 60.4%, Hb 126 g/L, PLT 276×10^9^/L; 5-month follow-up: hs-CRP 15 mg/L, ESR 62 mm/h, WBC 5.87×10^9^/L, NEUT% 47.3%, LYM% 45.6%, Hb 115 g/L, PLT 447×10^9^/L	Tofacitinib 3.5 mg twice daily, oral	After 2 months, marked clinical improvement with significantly reduced joint swelling and pain; no adverse events (infections or laboratory abnormalities)

DR, Digital Radiography; WBC, White Blood Cell; NEUT, Neutrophil; LYM, Lymphocyte; Hb, Hemoglobin; PLT, Platelet; MRI, Magnetic Resonance Imaging; CRP, C-reactive protein; ESR, Erythrocyte Sedimentation Rate; TNF-α, Tumor Necrosis Factor-alpha; IL-1β, Interleukin-1 beta; IL-4, Interleukin-4; IL-5, Interleukin-5; IFN-α, Interferon-alpha; IL-12p70, Interleukin-12 subunit p70.

### 2.2 Genetic analysis

**Figure 2:**
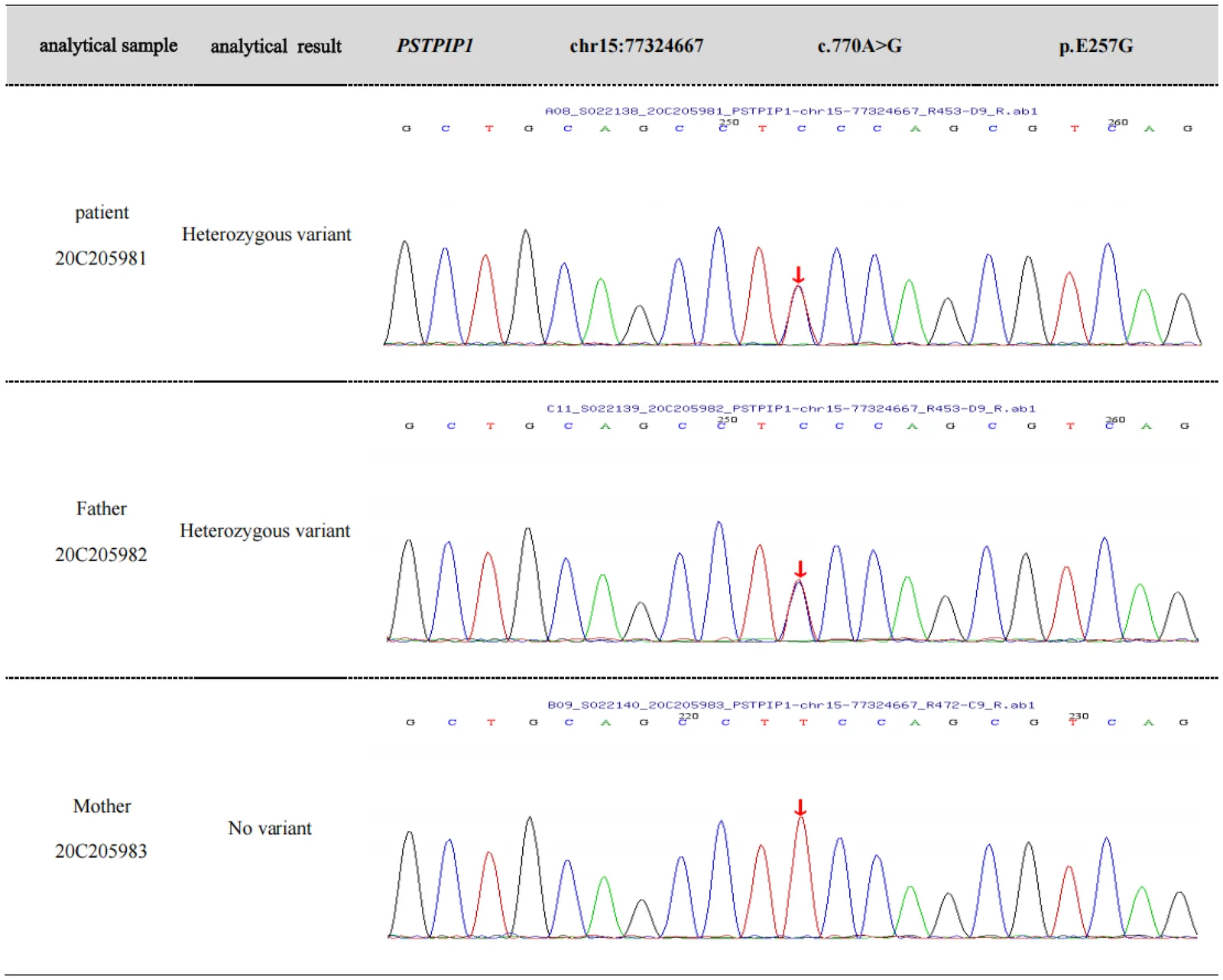
The genetic analysis. It shows the confirmatory Sanger sequencing analysis of PSTPIP1. Forward and reverse strand sequencing were both used for the analysis.

At age 7 (2021), trio whole-exome sequencing identified a heterozygous PSTPIP1 c.770A>G (p.Glu257Gly) variant. Segregation analysis showed the same variant in the father but not in the mother. The variant is absent from gnomAD (v4.0) and was classified as likely pathogenic (ACMG: PM1, PM2, PP3, PP5) based on its location in the F-BAR domain and previous reports of pathogenic alterations at codon 257 (p.E257K). Diagnosis of PAPA syndrome was confirmed. **Figure 2** showed the confirmatory Sanger sequencing chromatograms. The father (currently age 41) had experienced arthralgias in early childhood, but his joint symptoms completely resolved spontaneously after age 10, and he has since been asymptomatic without any treatment.

The p.E257G variant represents an extremely rare pathogenic change. A literature review of the Eurofever registry, the largest series of PAPA syndrome patients reported to date, identified this specific variant in only one of 16 patients, all of European descent (9). This report thus represented an Asian case of p.E257G-associated PAPA syndrome, providing important genotype-phenotype data across ethnic populations.

### 2.3 Treatment and follow-up

Following diagnosis, treatment with prednisone (0.5 mg/kg/day), methotrexate (15 mg/m²/week), and adalimumab (40 mg every other week) provided only partial and transient improvement. IL-1 inhibitors (anakinra 100 mg/day or canakinumab 2 mg/kg every 4 weeks) were not accessible owing to financial constraints. In February 2026, tofacitinib was initiated at 3.5 mg twice daily, a dose adjusted from the adult regimen of 5 mg twice daily based on body surface area. After 2 months of tofacitinib therapy, the patient showed significant clinical improvement: joint swelling and pain decreased markedly. Although hs-CRP and ESR are not specific indicators of inflammation, we observed that these laboratory parameters had also declined. No adverse effects (infections, laboratory abnormalities) were noted. No cutaneous lesions (acne or PG) have appeared to date.

However, the patient still had limited joint mobility. Radiography performed in March 2026 revealed structural damage: bone demineralization, articular surface osteophyte formation and roughening, joint space narrowing, epiphyseal margin roughening. Rehabilitation specialists considered the joint structural damage and advised against excessive rehabilitation training; the current priority was to prevent muscle atrophy. As of July 10, 2026, no recurrence of joint swelling and pain had been observed during follow-up. Long-term follow-up is ongoing.

## 3 Discussion

### 3.1 Diagnostic challenges

This case illustrated the diagnostic odyssey typical of PAPA syndrome. Data from the Eurofever registry reported a mean diagnostic delay of 18.8 years (range: 2 months to 50 years), with disease onset typically occurring in early childhood (mean age: 5.7 years) (9). A recent systematic review of 93 patients similarly reported a median diagnostic delay of approximately 19 years, with 62.5% of patients initially misdiagnosed with septic arthritis (10). In this case, although genetic identification was completed in January 2021, owing to insufficient physician experience at that time, the definitive diagnosis and targeted therapy were delayed until 2022–2026, leading to irreversible joint damage. The initial trauma and prolonged absence of cutaneous manifestations led to three unnecessary surgical debridements. This underscores the importance of considering autoinflammatory diseases in children with culture-negative, antibiotic-unresponsive recurrent arthritis, even in the absence of skin findings.

Chronic recurrent multifocal osteomyelitis (CRMO) should be included in the differential diagnosis. CRMO typically presents with bone pain and characteristic imaging findings, including osteolytic lesions on MRI and increased uptake on bone scintigraphy (11). Unlike PAPA syndrome, CRMO typically shows symmetric bone lesions and lacks the classic triad of arthritis, acne, and pyoderma gangrenosum. Other conditions requiring exclusion include septic arthritis, juvenile idiopathic arthritis, and other monogenic autoinflammatory syndromes such as DADA2 (deficiency of adenosine deaminase 2) and SAVI (STING-associated vasculopathy with onset in infancy) (12).

### 3.2 Pathogenicity of p.E257G

The p.E257G variant is located in the F-BAR domain (amino acids 1-308), which is critical for membrane localization and interaction with pyrin and other signaling proteins (7). Multiple lines of evidence support pathogenicity: (1) the variant is absent from population databases (gnomAD v4.0, TOPMed); (2) in silico predictions are deleterious (REVEL = 0.87, CADD = 24.8); (3) pathogenic variants at the same codon (p.E257K, p.E257Q) have been reported in multiple families with classic PAPA or PAMI phenotypes (13, 14); and (4) family segregation shows variable expressivity. The father, carrying the same mutation, exhibited only self-limited arthralgias in early childhood that resolved after age 10, demonstrating incomplete penetrance, consistent with PSTPIP1-associated disorders (8).

This case provided important ethnic-specific data on p.E257G pathogenicity. Previously reported cases were predominantly of European descent, as the Eurofever registry identified this specific variant in only one patient from an Italian cohort (9). By reviewing the literature (**Table 2**), we found that the present case represents the first Asian patient with this variant. The similar pathogenic mechanism across ethnicities supports the variant’s causative role regardless of genetic background.

**Table 2:** Characteristics of literature review.

Author(year/country)	Age of onset/gender/Age of diagnosis	HGVSc/HGVSp	Manifestations	Treatment	Treatment response and prognosis
Lindor NM et al. (1997/USA) (1)	4 years/Male/39 years	NA	arthritic changes in several joints, PG, and cystic acne	prednisone (60 mg daily), azathioprine (100 mg daily)	arthritis and PG partially improved
Cortis E et al. (2004/Italy) (22)	2 years/Male/22 years	c.904G>A/p.E302K	Recurrent pyogenic sterile arthritis; cystic acne	Glucocorticoids; cyclosporine A; etanercept 25 mg twice weekly	Initial response to steroids then resistance; etanercept induced rapid & sustained remission; acne resolved; mild hip motion limitation persisted
Dierselhuis MP et al. (2005/The Netherlands) (23)	16 years/Male/16 years	c.688G>A/p.Ala230Thr	Recurrent pyogenic sterile arthritis (triggered by minor trauma); mild acne; family history of PAPA syndrome	Intra-articular steroids (triamcinolone hexacetonide); anakinra 1 mg/kg/day s.c.	No response to intra-articular steroids; rapid and complete resolution of arthritis within 1–2 days after anakinra; ESR/CRP normalized
Brenner M et al. (2009/Germany) (24)	Childhood onset/Male/42 years	c.688G>A/p.Ala230Thr	Recurrent PG; recurrent erosive arthritis (knees, elbows, ankles); scarring acne	Topical tacrolimus; systemic glucocorticoids; anakinra 100 mg daily s.c.	Marked improvement in 5 days; complete ulcer healing in 1 month; arthritis and acne remained inactive; no relapse after treatment cessation
Tofteland ND et al. (2010/USA) (25)	2 years/Female/49 years	NA	Recurrent PG; recurrent inflammatory arthritis (elbow, ankle); flexion contracture; fever; family history of PAPA	Prednisone; etanercept 50 mg weekly	Poor response to high-dose steroids; progressive ulcers; etanercept led to sustained wound healing and clinical improvement
Schellevis MA et al. (2011/The Netherlands) (26)	3 years/Female/6 years	c.688G>A/p.Ala230Thr	Recurrent pyogenic sterile arthritis; fever; pustulosis	Antibiotics; ibuprofen; prednisone; topical corticosteroids	No response to antibiotics/ibuprofen; good response to prednisone; topical steroids effective for skin lesions
	18 years/Female/33 years	c.688G>A/p.Ala230Thr	Recurrent abdominal pain; acne; hidradenitis; arthritis; antiphospholipid syndrome; thrombosis; spontaneous abortion	Anakinra 100 mg daily s.c. (5 days); aspirin	Rapid resolution of fever/arthritis; aspirin effective for thrombosis
	16 years/Female/48 years	c.688G>A/p.Ala230Thr	Acne; arthritis; abscesses; sarcoidosis; proteinuria	Local antibiotics; prednisone	Local antibiotics effective for abscesses; response to prednisone unknown
Demidowich AP et al. (2012/USA) (8)	Infancy/Male/46 years	c.688G>A/p.Ala230Thr	Recurrent pyogenic sterile arthritis; PG; cystic acne	Plasmapheresis, thalidomide, dapsone, anakinra, tacrolimus, infliximab	Poor response to multiple agents; sustained remission with infliximab
	4 months/Male/10 years	c.748G>C/p.E250Q	Pathergy; pyogenic sterile arthritis; osteomyelitis; PG; recurrent otitis	Corticosteroids, etanercept, anakinra, infliximab, adalimumab	Good response to steroids, anakinra, adalimumab; no response to etanercept
	Infancy/Male/20 years	c.688G>A/p.Ala230Thr	Sterile abscesses; arthritis; cystic acne; PG	IVIG, colchicine, infliximab, methotrexate + prednisone + adalimumab	No response to prior agents; good response to combination therapy
	8 years/Male/18 years	c.688G>A/p.Ala230Thr	Recurrent pyogenic sterile arthritis; cystic acne	Isotretinoin, anakinra	Good control with anakinra; acne improved with isotretinoin
	1 month/Female/36 years	c.748G>A/p.E250K	Rash; lymphadenopathy; splenomegaly; thrombocytopenia; hemolytic anemia; pyogenic sterile arthritis; PG	Corticosteroids, cyclosporine, tacrolimus, anakinra, G-CSF	Partial response to cyclosporine/tacrolimus; no response to anakinra; died of sepsis
Caorsi R et al. (2014/Italy) (27)	18 months/Female/33 months	c.748G>C/p.E250Q	Recurrent pyogenic sterile arthritis; osteolytic bone lesion; periostitis	NSAIDs; prednisone; anakinra	Poor response to NSAIDs; good response to steroids; anakinra normalized inflammation and resolved bone lesions
Fathalla BM et al. (2014/Qatar/Jordan) (28)	6 months/Male/4 years	c.736G>A/p.D246N	Recurrent pyogenic sterile arthritis; vaccine-site abscess; no acne/pyoderma	Antibiotics; prednisone; naproxen; colchicine	Rapid response to steroids; no response to antibiotics/colchicine; biologic agents unavailable
Löffler W et al. (2017/Germany) (29)	8 years/Male/42 years	c.688G>A/p.A230Thr	Recurrent pyogenic sterile arthritis; severe cystic acne	Intra-articular steroids; oral steroids; anakinra; canakinumab; isotretinoin	Good response to steroids/anakinra/canakinumab for arthritis; acne controlled with isotretinoin
Guo YN (2020/China) (5)	6 years/Male/7 years	c.562 + 114C>G (novel)	Recurrent pyogenic sterile arthritis; family history; no acne/pyoderma	Naproxen; prednisone; methotrexate; tocilizumab	No response to conventional therapy; excellent response to tocilizumab; no flares during treatment
Wang Y et al. (2021/China) (20)	12 years/Female/49 years	c.356A>G/p.Y119C (novel)	PG; arthritis; joint deformity; keratitis; proteinuria; hematuria	Prednisone; methotrexate; cyclosporine	Rash, arthritis, and ophthalmic manifestations markedly improved
	24 years/Female/34 years	c.688G>A/p.A230T	Recurrent pyogenic sterile arthritis; PG; acne; oral ulcers	Leflunomide; diclofenac; short-course prednisone	Arthritis well-controlled; no recent PG flares
	2 years/Male/26 years	c.688G>A/p.A230T	Childhood-onset recurrent arthritis; fever; no acne/PG	Low-dose prednisone	Good response; no recent flares

HGVSc, human genome variation society coding; HGVSp, human genome variation society protein; PG, pyoderma gangrenosum; NA, not reported.

### 3.3 Therapeutic implications

IL-1 inhibitors are considered first-line therapy based on pathophysiology, and a growing body of evidence supports their efficacy (15, 16). However, cost barriers limit accessibility in many countries. This case provides preliminary, short-term evidence of clinical improvement with tofacitinib in a patient who could not afford IL-1 therapy.

We speculate that tofacitinib may inhibit JAK-STAT signaling downstream of the IFN-γ receptor, thereby reducing IFN-γ-induced pyrin expression. This could lead to decreased pyrin inflammasome activation and suppressed IL-18 secretion. By interrupting this pyrin–IL-18–IFN-γ loop, tofacitinib might attenuate the excessive inflammasome-driven inflammation characteristic of PAPA syndrome, independently of direct IL-1β inhibition (17, 18).

The marked improvement observed after 2 months suggests that JAK inhibition is a viable alternative for patients who cannot access IL-1 inhibitors. However, long-term follow-up is essential to assess the durability of response and safety, including monitoring for infections, laboratory abnormalities, and potential malignancy risk (19). Published data on tofacitinib use in PAPA syndrome remain limited, and this report contributes to the growing but still insufficient evidence base.

Nevertheless, this case also revealed a critical limitation: tofacitinib cannot reverse established structural joint damage. Despite achieving good inflammatory control (normalized CRP/ESR, resolution of joint swelling and pain), the patient was left with permanent mobility limitation due to bone demineralization, joint space narrowing, and osteophyte formation. This highlighted that while JAK inhibition is a viable alternative for inflammation control, it is not a substitute for early, aggressive intervention. The optimal window for preventing sequelae is likely early in the disease course, before irreversible bone changes occur.

### 3.4 Asian population data gap

A significant gap exists in PAPA syndrome literature regarding Asian populations. A recent systematic review of 93 patients with PAPA and PAPA-like syndromes revealed that the majority of reported cases originated from Western populations, while Asian patients remain markedly underrepresented (10). This disparity limited our understanding of genotype-phenotype correlations, treatment responses, and disease natural history across ethnic groups. In China, PAPA syndrome has been sparsely documented, with only a handful of cases reported to date (20, 21). Our case contributed to addressing this gap by reporting a Chinese pediatric patient with PAPA syndrome treated with tofacitinib, providing valuable data on disease presentation and treatment response in an under-represented population.

## 4 Conclusion

PAPA syndrome should be considered in children with unexplained, culture-negative, antibiotic-unresponsive recurrent arthritis, even in the absence of characteristic cutaneous manifestations. Early molecular diagnosis is essential to prevent unnecessary surgical interventions and prolonged morbidity. Preliminary observations from a 5-month follow-up suggest that tofacitinib may serve as an alternative for controlling inflammation in patients who lack access to IL-1 inhibitors due to economic or geographic constraints; however, longer-term follow-up is required to establish definitive conclusions regarding its efficacy and safety. And this case also demonstrated that tofacitinib cannot reverse established structural joint damage, underscoring the critical need for early, aggressive intervention to prevent irreversible sequelae. Once bony changes occur, the focus shifts to preserving muscle mass and preventing further functional decline rather than restoring mobility. To our knowledge, this report provide preliminary evidence of the potential efficacy of tofacitinib in pediatric patients with PAPA syndrome in China, and highlights the importance of timely treatment to prevent permanent disability.

## Data Availability

The original contributions presented in the study are included in the article/supplementary material. Further inquiries can be directed to the corresponding author.
